# Data for understanding trust in varied information sources, use of news media, and perception of misinformation regarding COVID-19 in Pakistan

**DOI:** 10.1016/j.dib.2020.106091

**Published:** 2020-07-30

**Authors:** Waqas Ejaz, Muhammad Ittefaq

**Affiliations:** a School of Social Sciences & Humanities (S3h), National University Science and Technology, Islamabad, Pakistan; b William Allen White School of Journalism and Mass Communications, University of Kansas, Lawrence, United States

**Keywords:** Covid-19 pandemic, Trust in information source, Misinformation, Pakistan, Online survey, Media use

## Abstract

The current data from 537 Pakistani millennials tell us about their trust in different information sources, the use of news media, and the perception of misinformation regarding COVID-19 in Pakistan. The dataset includes variables such as age, marital status, gender, social class, residential area, trust in the source of information, use of news media for coronavirus information, and perception of misinformation regarding COVID-19 in Pakistan. We fielded a survey from April 24 to May 12, 2020, via Qualtrics to obtain a convenient sample of younger and older adults in Pakistan. During this time, the number of new cases increased from 12,733 to 34,336. The surge took place despite the country being under a strict nationwide lockdown with the government relentlessly seeking the support of its policies from the people. This data may help scholars to understand how people of Pakistan interacted with different information sources, in comparison with other countries.

**Specifications Table**

**Table:** 

Subject	Infectious diseases, media and communication, information science
Specific subject area	Social science theories will be applied to understand the infectious disease data to examine the use of news media, trust in the source of information, and perception of misinformation regarding COVID-19.
Type of data	Tables
How data were collected	Online survey
Data format	To obtain the descriptive statistics, the raw data were analyzed with the help of R and RStudio. The authors have provided a raw version of the data file in Comma Separated Values (.CSV) format.
Parameters for data collection	There is no parameter used for data collection. Anyone could participate in the survey if they have an Internet connection.
Description of data collection	An online survey was administered through Qualtrics (one of the leading online sites to collect data). We used social networking sites, personal connections, and emails to collect representative data from Pakistani nationals currently living in Pakistan. Anyone who had access to the Internet could take the survey. The survey questionnaire is provided in supplementary files.
Data source location	Region: South Asia Country: Pakistan
Data accessibility	Repository name: Mendeley Digital Object Identifier:10.17632/6p4v8nssm2.3 Direct URL to data: https://data.mendeley.com/datasets/sd4239z35x/draft?a=55575b1ad647- 4e32-856b-5ef186fa4078 Mendeley data: Version 4

**Value of the data  **

1 Our dataset is relevant because it is the first data collected from Pakistani millennials (with an average age of 26 years) surveyed regarding their engagement with the media and (mis)information concerning COVID-19. The measures were adopted from The Reuters Institute for the Study of Journalism at Oxford University [1].
2 The data is particularly useful for public health officials, social scientists, and policymakers because it helps to understand how people tend to interact with the COVID-19 related information, and which sources are useful while communicating their respective messages.
3 The present data can be reused for an empirical study that intends to examine various characteristics of media use and trust in information sources in Pakistan, compared with other countries.
4 Due to the global health emergency, this dataset can be used to make informed decisions with regards to formulating crisis communication strategies in the wake of any future health crisis.
5 Amidst the pandemic, when people are eager to find more reliable and trustworthy information - to the best of our knowledge - it is the first data collected from Pakistan that examines people's perceptions toward sources of (mis)information regarding COVID-19.

## 1 Data description

The COVID-19 has proven to be a crisis with far-reaching global implications. The World Health Organization (WHO) declared it a pandemic and an international emergency in March 2020. Since the beginning of the outbreak, scholars from various fields such as public health, information communication and technology, risk perception, and social psychology started researching COVID-19 related issues. However, much of the focus has remained on Western countries where adequate research infrastructure already exists. Consequently, countries such as Pakistan have not been able to curate a dataset that can disentangle the complex human behavior regarding their interaction with COVID-19 related information [2]. Hence, this dataset, in addition to demographic variables, allows scholars to draw insights on trust in information sources, use of news media, and the perception of misinformation regarding COVID-19 in Pakistan.

Table 1 represents the socio-demographic variables. Amongst them, age and social status (0 = *low*, 10 = *high*) were a continuous scale variable, whereas, categorical variables that subsequently were recoded, i.e., gender (1 = *male*, 2 = *female*; marital status (1 = *in relationship*, 2 = *married*, and 3 = *single*), urban/rural (1 = *don't know*, 2 = *rural*, 3 = *urban*).

**Table 1:** Data summary of demographic variables.

Variable	Obs.	Mean	S.D.	Min.	Max.
**Age**	526	26.5	7.59	16	68
**Marital Status**	529	2.47	0.70	1	2
**Gender**	530	1.54	0.49	1	2
**Social class**	525	6.18	1.84	0	10
**Urban/Rural**	529	2.86	0.37	1	3

*Note:* The respondents could skip questions they did not want to answer. Therefore, the number of observations varies for each question.

Table 2 shows the descriptive statistics of the first measured variable in which respondents were asked on a scale from 1 (*no trust*) to 10 (*complete trust*) to assess the trustworthiness of each of the following sources of information regarding COVID-19.

**Table 2:** Descriptive statistics of people's trustworthiness.

Items	Obs.	Mean	S.D.	Min.	Max.
**Scientists**	531	8.10	1.98	0	10
**Government**	530	6.16	2.61	0	10
**Politicians**	530	3.87	2.68	0	10
**People I know**	529	5.39	2.68	0	10
**Traditional news (TV, newspaper, radio)**	530	5.59	2.74	0	10
**Facebook**	526	4.35	2.91	0	10
**Twitter**	526	4.71	3.02	0	10
**YouTube**	525	4.35	2.88	0	10

*Note:* The respondents could skip questions they did not want to answer. Therefore, the number of observations varies for each question.

To understand how people have used different sources in the last four weeks, respondents were asked to indicate which of the mentioned medium they have used in last month to get news related to the coronavirus summarized in Table 3.

**Table 3:** Descriptive statistics regarding the use of different news sources in the last month.

Items	Obs.	Mean	S.D.	Min.	Max.
**Online websites**	531	7.46	2.63	0	10
**TV**	532	6.43	3.15	0	10
**Newspapers**	529	3.30	3.29	0	10
**Facebook**	532	5.83	3.46	0	10
**Twitter**	532	3.82	3.65	0	10
**YouTube**	531	4.90	3.57	0	10

*Note*: The respondents could skip questions they did not want to answer. Therefore, the number of observations varies for each question.

Table 4 presents the descriptive regarding people's perceptions toward misinformation that they may have encountered regarding COVID-19 from multiple sources.

**Table 4:** Data description of people's perception of misinformation.

Items	Obs.	Mean	S.D.	Min.	Max.
**Scientists**	517	6.12	2.90	0	10
**Government**	513	5.65	2.57	0	10
**Politicians**	514	4.99	2.84	0	10
**People I know**	514	5.32	2.55	0	10
**Traditional news (TV, newspaper, radio)**	516	5.41	2.39	0	10
**Facebook**	516	4.96	2.79	0	10
**Twitter**	512	4.95	2.70	0	10
**YouTube**	510	4.97	2.79	0	10

*Note:* The respondents could skip questions they did not want to answer. Therefore, the number of observations varies for each question.

**Table 5:** Codebook and related questions.

Codebook	Questions
**Gender**	What is your gender?
**Age**	How old are you?
**Marital status**	What is your current marital status?
**Employment**	How would you describe your current employment status?
**Social class**	Please think of a ladder at top of which stands best off people and at bottom stands worst off people in Pakistan. Where do you see yourself on that ladder?
**Area of living**	Which of the following best describes the area you live in?
**Media use**	From 0 to 10, which, if any, of the following (online websites, TV, newspaper and so forth) have you used in the last month as a source to get coronavirus related news?
**Trust in sources**	From 0 to 10, how trustworthy would you say news and information about coronavirus from the following sources (scientists, politicians, TV, and so forth).
**Misinformation**	From 0 to 10, how much false or misleading information about coronavirus, if any, do you think you have seen or heard from each of the following sources (scientists, TV, Facebook and so forth) within the last month?

## 2 Experimental design, materials and methods

### 2.1 Data collection

The data collection took place between April 24 and May 12, 2020, via an online survey. In total, 537 Pakistani nationals aged 16–68 participated in the survey. The survey took on average 5–7 min to complete. Since the focus of our work was to collect representative data across age and gender only, therefore, we tried to distribute our study across all regions of Pakistan. Moreover, we used online sources such as WhatsApp, Facebook, and email to share the questionnaire. We used Qualtrics software provided by the University of Kansas, Lawrence, United States, to organize and distribute our survey.

Though the data collection was aimed to be representative across age and gender due to a large group of younger participants [3], most of the respondents were between 18 and 39 years old. This attribute increases the significance of the data. Because the past literature shows that young adults (24–39 years of age) are more likely to engage with multiple media platforms, and are more prone to use online media for consuming information, which is a relevant measure in this data, compared to the older population [4], [5], [6]. Furthermore, concerning gender, both males and females are equally represented in the dataset. We used R and RStudio to perform data analysis.

## Ethics statement

Before data collection, the instrument was reviewed and approved by the first author's university Institutional Review Board. The authors received informed consent from participants. Participation was voluntary, and they could withdraw from the survey at any point. As an ethical research team, we value the privacy rights of human subjects. Therefore, the data we submitted does not identify participants based on their responses. The survey did not collect any identifiable information from the participants.

## Credit author statement

**Waqas Ejaz** conceptualized and created the survey. **Muhammad Ittefaq** added the survey in Qualtrics and distributed it among participants. Both authors were involved in the data collection process. **Muhammad Ittefaq** wrote the paper. **Waqas Ejaz** reviewed and edited the final version.

## Declaration of Competing Interest

The research team did not receive financial support from any institutions. The authors declare that they have no known competing financial interests or personal relationships that could have appeared to influence the work reported in this paper.
